# The Implementation of a Doula Grant Program Directed at Families from Economically Vulnerable Backgrounds: A Process Evaluation

**DOI:** 10.1089/heq.2023.0264

**Published:** 2024-06-27

**Authors:** Samantha Wall, Kailey Snyder, Becky Baruth, Kara Foster

**Affiliations:** ^1^ Omaha Better Birth Project, Omaha, Nebraska, USA.; ^2^ University of Nebraska Medical Center, Omaha, Nebraska, USA.; ^3^ Omaha Better Birth Project, Omaha, Nebraska, USA.; ^4^ Nebraska perinatal Quality Improvement Collaborative, University of Nebraska Medical Center, Omaha, Nebraska, USA.

**Keywords:** doula, process evaluation, birth, grants

## Abstract

**Purpose:**

The purpose of this study was to evaluate the process and overall feasibility of a doula grant program for expectant families from economically vulnerable backgrounds.

**Design:**

A mixed-methods process evaluation framework was utilized to examine program feasibility and focused on constructs related to fidelity, dose delivered/dose received, reach, program satisfaction, and limited efficacy testing.

**Measures:**

Evaluation constructs were measured using a program tracking document. Program satisfaction and efficacy were examined through a mixed methodology approach utilizing doula surveys and birthing parent interviews.

**Analysis:**

Related to survey data, analysis focused on presenting descriptive counts and percentages related to the number of doulas that participated and clients served. Continuous variables were calculated as means and standard deviations and categorical data as counts and percentages. Qualitative data analyses were conducted using a structured deductive thematic approach.

**Results:**

The grant program was successfully implemented over an 18-month period, and the program had a high rate of fidelity to the grant processes developed by a Midwestern-based nonprofit organization. The program was satisfactory to doulas and parents, and the largest barrier was communication. A high incidence of prenatal anxiety among the birthing parents was reported. Thematic findings from the birthing parent interviews included the following: the grant application process was effective and easy, birth doulas were greatly valued, and a financial burden was lifted.

**Conclusion:**

This grant process can be replicated by other organizations seeking to fill a gap between doula services and the economically vulnerable.

## Background

The utilization of a birth doula is an evidence-based practice that has been shown to improve birth outcomes for both the parent and baby. However, few parents are able to utilize a doula, and some studies estimate this number to be as low as 6%.^1^ In addition, the value of a doula may not always be recognized due to limited understanding of the doula’s scope and role.^2^ Benefits of a birth doula are extensive and include reducing the incidence of cesarean sections, shortening the length of labor, reducing epidural and analgesic requests, increasing breastfeeding/chestfeeding initiation and continuation, increasing the birthing persons’ satisfaction of birth experience, reducing the incidence of postpartum mood disorders, and increasing new parents’ confidence in the care of their newborn, among others.^3–5^ Furthermore, a cost-effectiveness analysis indicated that doula support can produce a potential health care savings of $929–$1,047 per birth.^3^

As with any health care program, the use of a doula comes at a cost, a cost that can be a significant barrier to those without the means to pay for services. Moreover, doula services are not currently covered by public or private insurances in the state in which this program took place (Nebraska) making doulas even less accessible to many families. The only exception was TriCare insurance for military families during the time this evaluation took place, suggesting that this benefit was being underutilized.^6^ Importantly, 11.4% of United States families and 9.2% of Nebraska families fall under the official poverty rate.^7^ When a family is struggling to put food on the table or keep a roof over their head, essential services such as doulas and childbirth education become an unattainable luxury. Thus, a Nebraska-based nonprofit sought to fill the gap of missing services between the financially secure and insecure. Through the support of foundation grants, state Medicaid providers, and private donors, a grant program was developed to allow doula support to be provided to birthing parents with economically vulnerable backgrounds (e.g., low-income households, teen parents). This was a novel approach as access to doula care is often fragmented and reliant on self-research on behalf of the birthing parent or word of mouth referrals. However, the utilization of an outside entity serving as a mediator between a doula and a parent is novel and has not been investigated thoroughly. Thus, a process evaluation was warranted to better understand this innovative program. A process evaluation is a method used to assess the implementation and function of a program. Its primary purpose is to understand whether intended activities were executed as planned and support programmatic adjustments for enhanced success.^8^ Process evaluations are critical elements of program development to ensure appropriate resource allocation and to support program sustainability. Although several frameworks exist, one model that has been identified to support health promoting programs was developed by Saunders and colleagues (2005).^9^ Process evaluations often focus on elements related to feasibility, dose delivered, dose received, reach, recruitment, and program satisfaction. The value of process evaluations can be demonstrated by improved program effectiveness and quality, the ability to inform program scaling and replication, and increased accountability and transparency while also having the potential to empower stakeholders and allow them to contribute to decision making processes.^10–11^

The following narrative outlines a process evaluation that was conducted with 18 doula/parent dyads prior to the large-scale implementation of a doula grant program within a large Midwestern city. This process evaluation was conducted to help program administrators understand program strengths and barriers prior to program expansion. Thus, the purpose of this article is to describe the process evaluation findings and overall feasibility of a doula grant program for expectant families from economically vulnerable backgrounds.

## Methodology

### Doula Grant Program Model

A total of 12 doulas were contracted with the midwestern nonprofit to provide care to grant recipients prior to program launch. Doulas signed a contract that outlined a flat fee for their services. To be a doula partner of the organization, doulas were required to have a minimum of 1 year of experience and be certified in labor support. Participating doulas ranged in experience from 1 year to greater than 10 years. The average number of births previously attended by doulas was between 10 and 20 births; however, this varied widely per doula due to client base and personal time availability (e.g., doulas had additional employment).

Doula/organizational relationships were formed by previous contacts of the nonprofit’s Executive Director, a social media campaign, and word-of-mouth. The requirements of the doula/nonprofit contract included the following: providing their base birth doula services to at least one grant family per year at no more than $1,000 per family, providing at least 3–4 prenatal visits with their client, 1–2 postpartum visits, and completing a postpartum survey about the doula/awardee experience after each birth. Doulas were asked to be available to clients through multiple methods, including by phone, text, or email for questions and inquiries throughout the pregnancy and postpartum period. Importantly, $1,000 was selected after the nonprofit conducted an assessment of doula costs in the Midwestern United States. The assessment determined that doulas were being compensated at a rate between $700 and $1,200, and thus, a mid-level reimbursement of $1,000 was determined. Because this evaluation took place, the regional rate has increased and the nonprofit has increased its pay scale accordingly.

Participants were eligible to apply for a grant if they met the following criteria: participants must provide proof that they met the low-income criteria as per the Nebraska WIC guidelines OR be 19 years of age at least; must be planning to give birth in a local hospital; and must be between 15 and 32 weeks pregnant at the time the application is submitted. The grant application consisted of 18 items and obtained the following sociodemographic information: household income, age, housing status, race, ethnicity, household size, and gross household income. The pilot implementation lasted ∼18 months from Fall of 2021 to the end of the year in 2022. A total of 24 participants were sought.^9^ Nonprofit personnel (e.g., grants administrator) were responsible for confirming participant eligibility as per the criteria listed above prior to application review. Once eligibility requirements were met, grant awardees were selected on a first-come, first-served basis.

Awardees were informed of their grant through a congratulatory email which included their grant contract and a list of available doulas with their contact information. They had 2 weeks to provide documentation to the nonprofit related to proof of income, photo ID, and the signed contract. If an awardee did not provide these materials within 2 weeks of receipt, they were informed that they would be removed from the program. While selecting a program affiliated doula was not required, any nongrant partner doula had to meet the experience, and certification requirement to be approved and any costs incurred beyond the grant value were the responsibility of the awardee.

**FIG. 1.**
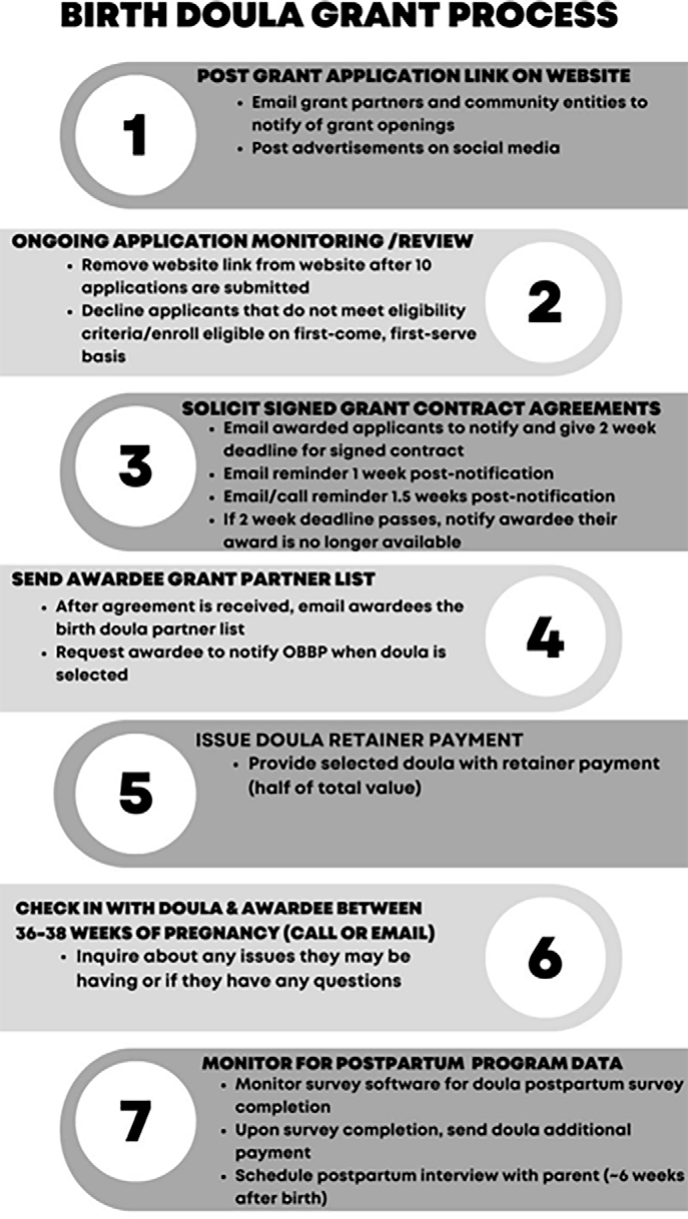
Grant Program Process.

Birth parent participation requirements included interviewing and selecting a birth doula, informing the nonprofit of their selected doula, committing to any agreed upon standards of practice between the doula and themselves (e.g., attending a number of prenatal visits, notifying the doula of labor onset), and completing a postpartum telephonic follow-up interview with program personnel. Doulas were responsible for reaching out to the nonprofit after their contract was signed to receive their retainer payment and again when the birth occurred. Prior to the final payment being received, doulas were required to submit an electronic survey on their experience. Additional details related to the grant program model are shown in Figure 1.

Importantly, after the first six families participated, the following three small process changes to the program model were made: (1) to reduce the burden on the birthing parent, follow-up interviews were discontinued; (2) birthing parents were given 1 month instead of 2 weeks to select and communicate with their doula; and (3) financial documentation proving financial status became required during the application process.

#### Evaluation methods

A process evaluation was used to assess participant recruitment, fidelity, program dose delivered/received, context, engagement, acceptability, and limited efficacy testing.^8^ The definitions utilized can be seen in Table 1. This program did not require institutional review board oversight as it was classified as an evaluative study based on the following definition: systematic collection of information about the activities, characteristics, and outcomes of programs to make judgements about the program, improve effectiveness, and/or information decisions about future program development (Patton, 2008).

**Table 1.** Outcome Definitions

Outcome	Definition
Fidelity	Program was delivered as intended
Dose Delivered	The amount of program that is delivered
Dose Received	The amount of program that is received
Reach	The number of mother/infant dyads reached
Satisfaction	Opinion of the program and its implementation
Limited efficacy-testing	Data collected to help inform intervention effect sizes needed for future programmatic efforts

#### Recruitment

Subjects were recruited through direct referrals from perinatal providers such as midwives, obstetricians, family practitioners, doulas, and childbirth educators. Recruitment also occurred through social media and email campaigns.

#### Data collection

All data materials were housed in *Google Docs* and *Airtable* and updated weekly. A tracking document was developed to collect data related to recruitment (e.g., grant applications received), due dates, delivery dates, etc. Doulas were asked to complete a post-program survey after the birth. The first six birthing parents were contacted by a program volunteer at 6 weeks postpartum to complete a telephonic interview. At the time of the phone call, the volunteer informed the parent that they would be asking questions about their experience with the doula grant program for program evaluation purposes. The parent was asked if they would be comfortable with the interview being audio recorded and told that their participation was voluntary and that they could stop at any time. If the parent agreed to continue by saying something to the effect of “yes” or “that’s fine” the interview commenced. Doulas were then sent a follow-up survey 18 months after their initial partnership with the nonprofit began to understand longitudinal influence of program engagement.

#### Assessment instruments

The post-program doula survey and birthing parent interview guide were developed in collaboration with a trained mixed methodologist (PhD) and two maternal child/health experts (MPH & PLMHP). The post-program survey completed by the doula was designed to assess fidelity and program satisfaction. The post-program doula survey included 15 items with a mix of multiple choice response and open-ended response and captured birth outcomes and delivery data. The survey questions are shown in Table 2. The longitudinal survey was a 3-item survey developed by the first author designed to assess how program engagement had influenced or shifted how doulas interact with client or the services they provide.

**Table 2.** Survey Questions

Post-program doula survey questions
Name of birthing client Name of doula Date of delivery Gestational age at birth What was the baby’s birth weight? What was the mode of delivery? What type of provider was present at the birth? To the best of your knowledge, did the client experience any of the following labor/delivery interventions? a. Induction b. Epidural c. Instrumental delivery d. Don’t know e. None of these To the best of your knowledge, did the client experience prenatal anxiety and/or depression? To the best of your knowledge, did the client experience any of the following postpartum complications? a. 1st, 2nd, 3rd, or 4th degree tearing b. Cardiac complications c. Hemorrhage d. Postpartum anxiety e. Postpartum depression f. Don’t know g. None of the above Did the birthing client intend to feed human milk after birth? a. If yes, were they able to feed human milk? Did you encounter any difficulties in meeting the needs of the grant family you served? a. If yes, please describe the issue(s) in as much detail as possible. Did the grant family you served fulfill their side of the contract with you? a. If no, please describe what happened in as much detail as possible. Would you recommend our organization to other families seeking pregnancy, childbirth, or postpartum resources? Please share any other comments about your experience with Omaha Better Birth Project and/or the family you served.
Post-program Follow-Up Survey
1. Have you seen an increase in use of your services as a result of participating in the doula grant program? 2. How has engaging with the doula grant program impacted the way you approach your clients? 3. How has participating in the doula grant program benefited you and/or your doula business?

The interview guide was developed to assess birthing parent satisfaction and engagement with the program. The interview guide included nine semistructured interview questions. The complete guide is shown in Table 3.

**Table 3.** Birth Parent Interview Guide

Postpartum interview questions
1. Did you have any difficulties accessing and/or applying for the birth doula grant?
2. Would you recommend our organization to other families seeking pregnancy, birth, and postpartum resources?
3. Did you experience any issues with your birth doula? If yes, please describe the issue(s) with as much detail as possible.
4. Many people describe an empowered birth as healing, safe, heard, and/or informed. How would you define an empowered birth? Would you consider your birth experience as empowered?
5. If you intended to provide human milk (either self-expressed or direct feed), were you able to meet this goal? If you are willing, please share about your experience.
6. Do you believe your birth doula played an important role in your pregnancy and birth experience?
7. Would you recommend a birth doula to other expecting families?

**Table 4.** Process Evaluation Outcomes

Outcome	Data source
Fidelity	1. Grant application • Twenty-seven applications received; seven lost to follow-up, two ineligible • Eighteen grant contracts signed • Recruited from friend/family referral (*n* = 4); social media (*n* = 3); organizational website (*n* = 1), health care provider (*n* = 6); other (*n* = 4) 2. Hundred percent post-program doula surveys completed
Dose Delivered/Dose Received	1. Twenty-seven grants received/18 awarded/18 contracts signed 2. Ninety four percent (*n* = 17) of birthing parents selected doula from organization network 3. Seventeen percent of families responded to organization’s check-in between 36–38 weeks of pregnancy 4. Doulas attended 100% of births
Reach	1. Doulas attended 100% of births

### Data Analysis

Related to survey data, descriptive statistics were determined. Continuous variables were calculated as means and standard deviations and categorical data as counts and percentages. Related to interview data, interviews were transcribed verbatim into a word document and uploaded into qualitative analysis software (NVivo 12). Qualitative data analyses (e.g., interviews and open-ended survey questions) were conducted using a structured thematic approach utilizing QSR Nvivo.^12^ First, open coding through a constant comparative analysis was used by a trained qualitative expert (PhD). Raw transcript data were read multiple times and split into sections. Axial coding then took place and the researcher identified connections across transcriptions, and categories were developed. Categories were reviewed again and collapsed based upon similarities. Categories were combined once more, and an overarching theme was identified based on categorical similarities. A student investigator reviewed all categories and themes and discussed discrepancies with the initial investigator. Discussions occurred until consensus was reached, and qualitative analysis was deemed complete.

## Results

### Grant Process Outcomes

During the program implementation period, 27 applications were received with seven individuals being lost to follow-up and two being ineligible. A total of 18 grant contracts were signed with recruitment occurring from friend/family (*n* = 4), social media (*n* = 3), the organization website (*n* = 1), a health care provider (*n* = 6), or another unidentified source (*n* = 4). Additional process details can be seen in table 4 below.

### Program Satisfaction

The doula post-program survey (*n* = 18), grant participation interviews (*n* = 4), and doula longitudinal follow-up surveys (*n* = 8) were utilized to assess program satisfaction. Overall, doula/parent dyads were extremely satisfied with their experience with the grant process. Specific to the doula post-program survey, all doulas reported that their grant families fulfilled their side of the grant contract. When asked if they encountered any difficulties in meeting the needs of the grant families served, all but two doulas said no. For instance, one doula noted, “*Nope! Everything was smooth sailing.*” The two doulas that experienced challenges reported barriers related to communication. For instance, “hard to reach at times, [the] husband worked nights.” When asked to share additional comments about the grant process, all doulas had positive perceptions of the experience. One noted, *“it’s an honor to be a part of this.”* Another stated, *“Y’all are doing great things out here! I have partnered with multiple organizations doing similar work/offering similar funding and yours was by far the smoothest process for both the client and myself. Much gratitude.”* All doulas (100%) reported that they would recommend the nonprofit to other families seeking pregnant, childbirth, or postpartum resources.

Specific to the post-program parent interviews (*n* = 4), three overarching themes were identified as follows: (1) the grant application process was effective and easy, (2) birth doulas were greatly valued, and (3) a financial burden was lifted. All awardees had positive perceptions of the grant application process, and there were no recommendations for improving the process. Regarding the doulas’ value, birthing parents reported that their doula played an important role in their pregnancy and birth experience. Doulas were reported to provide resources, education, and support throughout the pregnancy and during the birth. One parent reported, *“She gave me a lot of information that I didn’t know about as far as medical procedures and things that go along with being pregnant. When I found out I was breech, she gave me a few ideas to help before I got too close to birth and then during labor she was just very helpful. She guided me very well and helped me out with whatever I really needed that day. It was simple.”* All parents reported that the monetary component of the grant itself was a huge component in their satisfaction with the program. Parents stated that they would not have had the opportunity to utilize a doula without this program. One parent stated, “*It helped a lot financially. I knew I wasn’t going to be able to afford this, that was really one of the biggest things.*”

Finally, doulas were sent a 3-question survey after they had been engaged with the program for at least 18 months. When asked if they had seen an increase in use of their services as a result of being a part of the grant program, 6 out of 8 reported yes (75%). When asked how engagement in the grant program had impacted the way they approach their clients one primary theme emerged. Doulas reported that engagement in the program has increased their ability to support birthing parents of diverse backgrounds. For example, one doula noted “*I have worked with many different types of personalities, ethnicities and family dynamics and have learned from all of them to better know how I give my services.*” When doulas were asked how participating in the doula grant program has benefited them or their business, two primary themes emerged. The first included an increase in referrals. Several doulas reported that their engagement in the grant progress had made it easier to connect with new clients. One doula reported, *“Just by word of mouth or friend referrals, it definitely helps knowing people can find me easier.”* The other identified theme was related to enhanced ability to live out personal values. Doulas reported that their experience with the grant program better helped them to serve the population they most wanted to and demonstrate values that they found important. One doula stated, “*It enables me to support clients in my preferred population.”* Only one doula did not report a difference due to program engagement. This doula noted, “*at this time it [program engagement] has not [been beneficial]. It is neutral for my business, not negative in anyway but I haven’t seen a measurable difference.*”

### Limited-Efficacy Testing

To begin to evaluate program efficacy, birth outcome information was assessed. The following table describes the pilot cohort’s birth experiences as per the doula post-birth survey. The majority of parents had a vaginal birth (66.8%) and had an obstetrician as their health care provider (66.8%). Doulas reported that 50% of participants had self-reported prenatal anxiety although a formal diagnosis may not have been present. Of the participants, 16.6% had some degree of tearing, and 77.8% successfully fed human milk after delivery. Additional findings are shown in Table 5.

**Table 5.** Grant Recipient Birth Outcomes

Outcome	Mean (SD)
Gestational Age at Birth (weeks)	38.9 ± 1.3
Birth Weight (lbs)	7.1 ± 1.3
Mode of Delivery	N (%)
Vaginal	12 (66.8%)
Unplanned Cesarean	5 (27.7%)
Planned Cesarean	1 (5.5%)
Type of Provider	
OBGYN	12 (66.8%)
Midwife	5 (27.7%)
Family Practitioner	1 (5.5%)
Labor/Delivery Interventions	
Induction	5 (27.7%)
Epidural	6 (33.3%)
Induction & Epidural	3 (16.6%)
None	3 (16.6%)
Unsure	1 (5.8%)
Experienced prenatal anxiety	
Yes	9 (50.0%)
No	8 (44.4%)
Unsure	1 (5.6%)
Postpartum complications	
1^st^, 2^nd^, 3^rd^, or 4^th^ degree tearing	3 (16.6%)
Hemorrhage	1 (5.5%)
Postpartum Anxiety	1 (5.5%)
None of the above/unsure	13 (72.2%)
Intended to feed human milk	
Yes	16 (88.9%)
No	2 (11.1%)
Fed human milk after delivery	
Yes	14 (77.8%)
No	2 (11.1%)
Unsure	2 (11.1%)

## Discussion

This process evaluation determined the doula grant program to be feasible and satisfactory for birthing parents and doulas prior to larger scale implementation. Next steps will focus on developing a database and procedures to capture real-time health information for participants upon program expansion. Parent/infant sociodemographic, health history, and birth outcomes will be tracked and analyzed to better understand the reach and impact doula grants can have on parent/infant health. Our model is unique due to the inclusion of a third party organization serving as a mediator between the doula and the grant recipient. Doulas often rely on word-of-mouth and health care provider referrals to develop their client base. The inclusion of a nonprofit to serve as the bridge to the parent/doula dyad provides an additional avenue to expand the utilization of doulas. Our findings even suggest that engagement in such a program could result in more client referrals for doulas, while allowing doulas to work with more diverse populations. Barriers identified during the process evaluation were largely related to nonprofit communication with grant families after the contract was signed. Our reliance on technology in this process (e.g., phone/email check-ins) could be a burden to individuals with limited technological literacy or language barriers. Offering linguistically appropriate in-person or phone assistance may help mitigate these barriers. Furthermore, reducing organizational engagement after the contract is signed may be valuable (i.e., removing the organizational check-in between 36–38 weeks of pregnancy). Facilitators of this process include the network of doula partners that was developed prior to program launch. Hiring a doula can be a very personal and daunting choice, allowing families autonomy over their selection, and reducing the burden of having to go find these individuals was beneficial. An additional success for this program was the utilization of our tracking table through *Airtable.* Utilizing *Airtable* for administrative tracking of the application process and outcomes was easy to use and provided at-a-glance information. The technology allowed program administrators to house all the program data and information in one location at a minimal cost.

Related to the birth outcome data, although sample size limits generalizations related to efficacy data collected, the high incidence of self-reported anxiety suggests that a focus on doula support of mental health may be valuable. Future research should examine how previously developed interventions to address perinatal mental health can be adapted to be disseminated by doulas. In addition, the post-program doula questionnaire could include constructs of social support theory to better capture how birthing parents are obtaining vital support such as informational support, appraisal support (e.g., encouragement, praise), emotional support, and instrumental support (e.g., help with errands, childcare) related to the birthing process.^13^ This would help to better understand the gaps that are experienced by birthing parents and their families and potentially allow doulas to serve as mediators to community resources.

## Conclusion

Utilizing a nonprofit organization as a third party entity to disseminate a doula grant program to individuals from economically vulnerable populations is a feasible strategy that could be replicated by other organizations within the United States. This program benefited from its ability to reduce administrative burden on the doula by managing all payment processes and contract requirements. Furthermore, the program allowed birthing parents that otherwise may not have had access to doula services to receive them. Future programmatic efforts should focus on developing strategies to track demographics and health information to better understand the impact a doula grant can have on the parent/infant dyad.
